# Molecular Motors in Blood–Brain Barrier Maintenance by Astrocytes

**DOI:** 10.3390/brainsci15030279

**Published:** 2025-03-06

**Authors:** Ana Filipa Sobral, Inês Costa, Vanessa Teixeira, Renata Silva, Daniel José Barbosa

**Affiliations:** 1 Associate Laboratory i4HB—Institute for Health and Bioeconomy, University Institute of Health Sciences—CESPU, 4585-116 Gandra, Portugal; 2 UCIBIO—Applied Molecular Biosciences Unit, Toxicologic Pathology Research Laboratory, University Institute of Health Sciences (1H-TOXRUN, IUCS-CESPU), 4585-116 Gandra, Portugal; 3 Associate Laboratory i4HB—Institute for Health and Bioeconomy, Faculty of Pharmacy, University of Porto, 4050-313 Porto, Portugal; inessilvacosta@hotmail.com (I.C.); rsilva@ff.up.pt (R.S.); 4 UCIBIO—Applied Molecular Biosciences Unit, Laboratory of Toxicology, Department of Biological Sciences, Faculty of Pharmacy, Porto University, 4050-313 Porto, Portugal; 5 i3S—Instituto de Investigação e Inovação em Saúde, Universidade do Porto, 4200-135 Porto, Portugal; vanessa.teixeira@i3s.up.pt; 6 ICBAS—Instituto de Ciências Biomédicas Abel Salazar, Universidade do Porto, 4050-313 Porto, Portugal; 7 UCIBIO—Applied Molecular Biosciences Unit, Translational Toxicology Research Laboratory, University Institute of Health Sciences (1H-TOXRUN, IUCS-CESPU), 4585-116 Gandra, Portugal

**Keywords:** molecular motors, kinesins, dynein, myosins, intracellular transport, junctional components, astrocytes, blood–brain barrier

## Abstract

The blood–brain barrier (BBB) comprises distinct cell types, including endothelial cells, pericytes, and astrocytes, and is essential for central nervous system (CNS) homeostasis by selectively regulating molecular transport and maintaining integrity. In particular, astrocytes are essential for BBB function, as they maintain BBB integrity through their end-feet, which form a physical and biochemical interface that enhances endothelial cell function and barrier selectivity. Moreover, they secrete growth factors like vascular endothelial growth factor (VEGF) and transforming growth factor-beta (TGF-β), which regulate tight junction (TJ) proteins (e.g., claudins and occludins) crucial for limiting paracellular permeability. Molecular motors like kinesins, dynein, and myosins are essential for these astrocyte functions. By facilitating vesicular trafficking and protein transport, they are essential for various functions, including trafficking of junctional proteins to support BBB integrity, the proper mitochondria localization within astrocyte processes for efficient energy supply, the polarized distribution of aquaporin (AQP)-4 at astrocyte end-feet for regulating water homeostasis across the BBB, and the modulation of neuroinflammatory responses. Moreover, myosin motors modulate actomyosin dynamics to regulate astrocyte process outgrowth, adhesion, migration, and morphology, facilitating their functional roles. Thus, motor protein dysregulation in astrocytes can compromise BBB function and integrity, increasing the risk of neurodegeneration. This review explores the complex interplay between astrocytes and molecular motors in regulating BBB homeostasis, which represents an attractive but poorly explored area of research.

## 1. Introduction

The blood–brain barrier (BBB) is an essential structural and functional barrier for maintaining central nervous system (CNS) homeostasis [1,2]. Particularly, it plays a critical role in protecting the brain from potentially harmful substances while allowing the selective transport of nutrients and molecules required for brain homeostasis [1].

The BBB’s integrity relies on complex interactions between different cells, including endothelial cells and astrocytes. Endothelial cells are the fundamental structural components of the BBB. Their spatial organization, with tight and adherens junctions (TJs and AJs, respectively), restricts openings compared to the fenestrations usually found in peripheral capillaries, thereby limiting the diffusive movement of molecules to the brain [3]. Astrocytes play multiple roles in the CNS, including modulation of synaptic activity, control of axonal growth, immune response, and involvement in advanced cognitive processes such as memory [4,5]. Moreover, through extensive interactions with the brain vasculature, astrocytes support the integrity of the BBB [6]. The astrocytic end-feet enwrap blood vessels, forming a physical and biochemical interface that supports endothelial cells and enhances the selective permeability of the barrier [7]. Moreover, astrocytes secrete several growth factors and signaling molecules, such as vascular endothelial growth factor (VEGF) [8] and TGF-β [9]. These factors regulate the expression and function of TJ proteins like claudins and occludins, which are critical for limiting paracellular permeability [10,11]. Thus, in astrocytes, the transport and release of these molecules depend on efficient vesicular trafficking mechanisms orchestrated by molecular motors.

Molecular motors are proteins that convert chemical energy from adenosine-5′-triphosphate (ATP) hydrolysis into mechanical work, driving the movement of organelles, vesicles, and other cellular components along cytoskeletal tracks [12]. In eukaryotic cells, the two primary types of molecular motors are kinesins and dyneins, which travel along microtubules, and myosins, which move along actin filaments [12,13]. These motors play fundamental roles in a variety of cellular processes, including intracellular transport, cell division, and organelle positioning [12,13,14,15,16,17]. In the context of astrocytes, molecular motors are essential for mediating the transport of lipids [18], proteins, and organelles [19], enabling the cells to respond to external stimuli and maintain their supportive functions.

Other evidence also suggests that dysregulation of molecular motor function can contribute to BBB breakdown and CNS pathologies [20,21]. Several neurodegenerative diseases are characteristically associated with mutations or malfunctions in motor proteins [22,23,24,25,26,27,28]. Such perturbations can impair vesicular trafficking, mitochondrial transport, and overall cellular homeostasis, leading to compromised BBB integrity. Thus, understanding the role of molecular motors in astrocytes offers valuable insights into the mechanisms involved in BBB maintenance.

This review aims to explore the role of molecular motors in maintaining BBB integrity and function, with a focus on their involvement in astrocytic functions such as vesicular trafficking, endocytosis, the regulation of TJ proteins, and astrocyte morphology.

## 2. Overview of the Blood–Brain Barrier

The BBB is a critical structure responsible for maintaining the homeostasis of the CNS. It acts as a selective barrier between the blood and the brain, controlling the entry and exit of molecules, ions, and cells. Thus, it is essential for controlling blood flow, protecting the brain from toxins and pathogens, and ensuring that essential nutrients and molecules reach the CNS [2].

The existence of the BBB was first hypothesized by Paul Ehrlich in 1885. He noticed that the intravenous injection of dyes resulted in strong staining of peripheral organs, while the brain and spinal cord remained unstained [29]. This hypothesis was further corroborated by Lewandowski, who concluded that the entry of some molecules into the brain is prevented by brain capillaries [30]. Over the 20th century, electron microscopy and molecular biology studies have revealed that the BBB has indeed a complex cellular and molecular composition [2], rather than just a tight monolayer of endothelial cells.

### 2.1. Structure of the Blood–Brain Barrier

**Figure 1:**
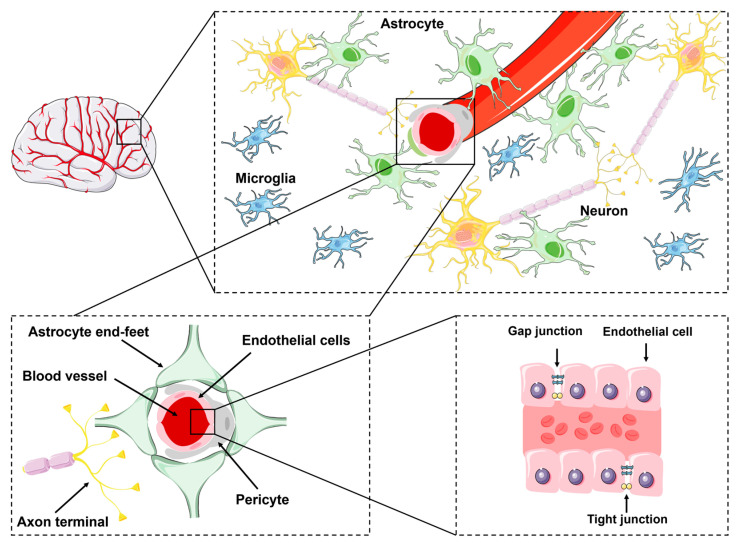
Schematic representation of the cellular components of the blood–brain barrier (BBB) and their interactions with neural and vascular elements at the neurovascular unit (NVU). (**Top right**): Zoomed-in view of the NVU, showing key cellular components, including pericytes (grey), astrocytes (green), neurons (yellow), microglia (blue), and a blood vessel (red). (**Bottom left**): Cross-sectional view of a blood vessel (red) and surrounding cellular elements [endothelial cells (pink), astrocyte end-feet (green), a pericyte (grey), and an axon terminal (yellow)]. (**Bottom right**): Endothelial tight and gap junctions responsible for limiting the permeability of the BBB. The illustration was created using images from Servier Medical Art.

Structurally, the BBB is primarily composed of endothelial cells that form the limits of blood vessels in the brain, with contributions from other cell types, including pericytes, vascular smooth muscle cells, astrocytes, neurons, microglia, and a basement membrane. These components form a complex network, collectively called the neurovascular unit (NVU), which is responsible for supporting proper brain function [3,31] (Figure 1).

#### 2.1.1. Endothelial Cells

Endothelial cells form the basic structural component of the BBB (Figure 1). These cells play crucial roles in various biological processes, such as forming a protective barrier, transporting micronutrients and macronutrients, mediating receptor-based signaling, facilitating leukocyte movement, and regulating osmotic balance [32].

In peripheral tissues, endothelial cells are characterized by the presence of fenestrations, which facilitate molecular exchange [33]. Moreover, the presence of gap junctions, which act as intercellular channels, allows for direct communication between adjacent endothelial cells and between endothelial and vascular smooth muscle cells [34]. In mammals, vascular gap junctions are composed of connexin (Cx) proteins [35,36]. By facilitating the exchange of ions and small molecules (<1000 Da) between cells, gap junctions regulate various physiological processes, including vascular tone, coordination of vasodilatory signals, modulation of endothelial permeability, and control of cellular stiffness [35,37].

At the BBB, gap junctions are present between astrocytes, pericytes, and endothelial cells, playing a crucial role in maintaining tight control and transmitting electrical signals necessary for neurovascular coupling [38,39]. In fact, among the different connexins, Cx43 is the most extensively expressed and investigated in astrocytes [36] and has been shown to interact and colocalize with zonula occludens-1 (ZO-1), an essential TJ protein that helps regulate gap junction formation between endothelial cells [39]. Gap junctions also enable direct metabolic and electrical communication between astrocytes and neurons [38]. In mice, the absence of astrocyte gap junctions is linked to extensive cell vacuolation [40]. Moreover, Cx43 levels decrease with aging, leading to BBB leakage and cognitive decline [41], suggesting a key role for gap junctions in BBB maintenance and function. In support of this observation, Cx43 global knockout leads to early postnatal death [42].

Unlike peripheral endothelial cells, brain endothelial cells lack fenestrations, or small openings. This is due to the presence of TJs and AJs that connect adjacent endothelial cells, which are critical for maintaining the barrier’s integrity (Figure 1). Tight junctions in brain capillaries are 50–100 times tighter than those in peripheral capillaries, which limits the passive movement of molecules into the brain, leading to a significantly higher trans-epithelial electrical resistance (TEER) in these blood vessels [43].

Tight junction proteins include claudins, occludins, ZO-1, and junctional adhesion molecules, while AJs are primarily formed of vascular endothelial (VE) cadherin molecules. Together, these components create a nearly impermeable seal that restricts paracellular transport, or the movement of substances between cells [3]. Impairment in the regulation of endothelial cell junctional proteins leads to the loss of BBB integrity, enabling systemic entry into the brain, which may induce swelling or neurotoxicity.

Claudins are key structural proteins in TJs, where they create a primary seal by binding to claudins on adjacent endothelial cells through homotypic interactions [44]. These claudins are connected to cytoplasmic proteins like ZO-1, ZO-2, and ZO-3 through their carboxyl-terminal ends, reinforcing the junctional complex. The interactions between claudins, both homophilic and heterophilic, promote close adhesion between cell monolayers, thereby maintaining the integrity and selective permeability of the BBB [45]. Claudin-3, claudin-5, and claudin-12 are thought to contribute to elevated TEER [46,47]. Indeed, claudin-5 has an important role in BBB function and TJ development, and some studies demonstrated that removal of embryonic claudin-5 (in a mouse model) promoted early postnatal brain swelling and death [48]. Occludin is a 60–65 kDa protein with four transmembrane domains, a short N-terminal cytoplasmic domain, and a long C-terminal cytoplasmic domain. The two extracellular loops of occludin and claudin from adjacent cells create the paracellular barrier within TJs, while the long C-terminal cytoplasmic domain of occludin is directly connected to ZO proteins (namely, ZO-1) [49]. Its principal role seems to be TJ regulation [32]. Membrane-associated guanylate kinase proteins are supportive elements for the transmembrane components of TJs. These proteins have multiple binding domains that promote protein–protein interactions, allowing them to cluster protein complexes at the cell membrane [50]. The primary proteins involved in TJs are ZO-1, ZO-2, and ZO-3. ZO-1 is predominantly expressed in endothelial and epithelial cells and contributes to TJ assembly by linking transmembrane TJ proteins to the actin cytoskeleton [51]. When ZO-1 is lost or dissociates from the junctional complexes, it correlates with increased barrier permeability [52]. Junctional adhesion molecules (JAMs), including JAM-A, JAM-B, and JAM-C, are found in cerebral endothelial cells and participate in the development and preservation of TJs [32].

Regarding AJs, cadherin molecules seem to join the actin cytoskeleton via intermediary proteins, such as catenins, to form adhesive contacts between cells [53]. Adherens junctions are formed through homophilic interactions between the extracellular domains of calcium-dependent cadherins on the surfaces of neighboring cells. The cytoplasmic domains of these cadherins connect with submembrane plaque proteins, such as β- and γ-catenin, which in turn link to the actin cytoskeleton via α-catenin [53].

The brain endothelial cells that form the BBB exhibit very low levels of transcytosis, reducing the movement of large proteins and other molecules across the cells into the brain [54]. These characteristics are critical for maintaining the internal environment of the brain and protecting it from fluctuations in the external environment that could disrupt neuronal function. Additionally, brain endothelial cells have a great number of mitochondria, enhancing the energy potential and the active transport of nutrients into the brain [55].

#### 2.1.2. Pericytes

Pericytes are mural cells that envelop capillary blood vessels on their abluminal side, sharing a basement membrane with endothelial cells (Figure 1). They establish direct synaptic-like peg–socket focal contacts with endothelial cells through N-cadherin and conexins. This close physical location between pericytes and endothelial cells enables the signaling between them, playing a role in vessel formation and maturation, pericyte coverage, and pericyte recruitment [56]. Additionally, this interaction allows the exchange of metabolites, ions, and second messengers between the two cell types [57]. Pericytes also appear to be responsible for maintaining BBB integrity, microvascular stability, angiogenesis, and the phagocytosis of toxic metabolites [57].

#### 2.1.3. Astrocytes

Astrocytes are a type of glial cell and the most abundant cells in the CNS, performing numerous physiological and biochemical roles, including maintaining ionic homeostasis in the extracellular environment, compartmentalizing neural tissue, regulating pH, mediating communication between neurons and the vasculature, and facilitating neurotransmitter uptake and processing by supplying energy-rich substrates to neurons. Additionally, astrocytes also have an important role in maintaining BBB integrity and function, protecting the brain against neurotoxins [58] (see Section 3 for a detailed discussion on the role of astrocytes in maintaining BBB integrity).

#### 2.1.4. Microglia

Microglia are a type of neuroglial cell that support neurons by providing immune defense, engulfing harmful foreign particles, repairing injured brain tissue, and participating in extracellular signaling. Additionally, microglia can regulate TJ expression, thereby enhancing the integrity and functionality of the BBB [59]. Some evidence also suggests that microglia support neovascularization of the CNS, providing a structural framework for the growth and development of blood vessels [60].

#### 2.1.5. Neurons

Neurons are the principal units of the brain and CNS and are responsible for rapid communication of information [61]. They play a crucial role in maintaining and regulating the BBB by upregulating the expression of key proteins such as TJ and AJ proteins [62]. Additionally, neurons seem to interact with other components of the NVU, contributing to the regulation of cerebral blood flow and microvascular permeability [63].

#### 2.1.6. Basement Membrane

The basement membrane, an extracellular matrix, provides structural support to the cells of the NVU and serves as a critical hub for intracellular communication and signaling pathways among these cells [64]. This structure is composed of diverse molecules, namely, fibronectin, type IV collagen, laminin, nidogen, proteoglycans, sulfate, and other glycoproteins. Interactions between the basement membrane and its associated cells are mediated by two types of matrix transmembrane receptors: dystroglycan and integrins [65]. Moreover, these interactions link the extracellular matrix to the cytoskeleton and activate signaling cascades that promote cell proliferation, migration, and survival. They also facilitate the activation of growth factors, thereby regulating the barrier function [66].

### 2.2. BBBs Across Different Animal Species

The cellular and structural organization of the BBB varies across different animal species, reflecting evolutionary adaptations to their neurological, metabolic, and environmental needs. However, the fundamental function of the BBB (controlling the passage of substances between the blood and the CNS) is conserved across vertebrates [67]. Similar to mammals, including humans, rodents, and primates, birds and reptiles also possess a BBB with TJs and astrocytic end-feet, but they allow higher molecular exchange [67,68,69]. Similar to other vertebrates, the NVU in amphibians and fish is composed of endothelial cells connected by TJs, glial cells, neurons, pericytes, and a basement membrane [70,71]. By contrast, in invertebrates, such as Drosophila, the outer cell layer of the BBB consists of a thin, discontinuous monolayer of perineurial glial cells [72,73].

Despite these similarities, the evolutionary complexity of the BBB has increased with the advancement of the CNS, driven by factors such as neural circuit complexity, increased brain size and longevity, and increased metabolic demands. As organisms developed to have larger and more complex brains, it became necessary to have a more selective and tightly regulated BBB [71]. Thus, the complexity of the BBB is essential for multiple purposes, including neuroprotection against xenobiotics and pathogens, to maintain the ion balance necessary for synaptic transmission, selective transport of key nutrients (e.g., glucose and amino acids), and to limit immune cell infiltration to prevent neuroinflammation [49]. Overall, these factors contribute to the efficiency and longevity of the nervous system, demonstrating why evolution favored a highly specialized BBB structure in more advanced species.

### 2.3. In Silico Models of NVU Dynamics in the BBB

In silico models are valuable tools for complementing experimental research and exploring complex biological contexts that are often challenging or inaccessible through the current in vitro and in vivo methods. An increasing number of mathematical modeling studies, using different approaches, are investigating the intricate cellular network dynamics within the BBB and providing important insights into the cellular interactions at this interface [74].

Shityakov and Förster [75] provided a comprehensive review of computational methods applied to understand BBB dysfunctions and disease-related impacts on the CNS at molecular, cellular, tissue, and organ scales. At the molecular and cellular levels, simulations unveiled the involvement of mutated or misfolded TJ proteins, receptors, and BBB transporters in cellular trafficking and their relation to pathological conditions, such as Alzheimer’s disease. At the tissue and organ levels, models enriched with multiple raw data sources from in vitro and in vivo studies, including histopathological, omics, and imaging data, explored how BBB injury and leakage affect the overall brain tissue morphology and interconnected functioning. Additionally, several mathematical models have been developed to investigate neuron–glia interactions within tripartite synapses: presynaptic neurons, synaptic terminals, postsynaptic neurons, and astrocytes. Simulations were able to replicate diverse neuronal population behaviors, including regular spiking, demonstrating that astrocytes modulate neuronal spiking activity, thereby influencing neuronal behavior [76,77]. Computational models have also described the impact of neuromodulators, such as calcium, glutamate, and noradrenaline, on neuron–glial interactions and synaptic plasticity [78]. Finally, models dedicated to investigating the influence of the BBB microenvironment’s physical properties and the role of the cytoskeleton and its molecular motors in NVU cells are emerging as important avenues to help understand the functioning of this complex biological system [79].

## 3. Role of Astrocytes in Blood–Brain Barrier Maintenance

**Figure 2:**
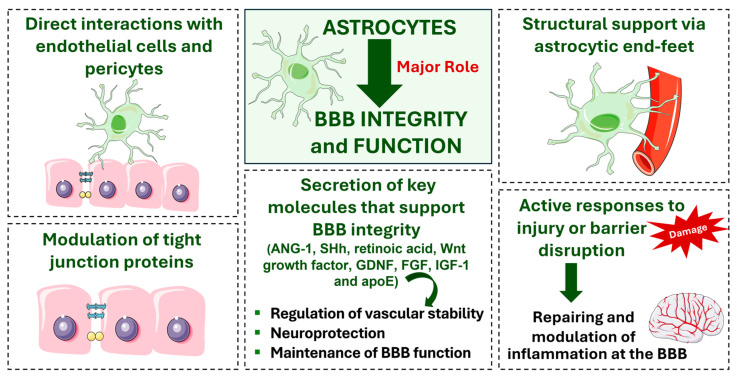
Role of astrocytes in maintaining blood–brain barrier (BBB) integrity and function. Astrocytes support the BBB through direct interactions with endothelial cells and pericytes, structural support via end-feet, modulation of tight junction proteins, and secretion of key molecules that regulate vascular stability, neuroprotection, and BBB maintenance. Additionally, astrocytes respond to injury by repairing and modulating inflammation at the BBB. ANG-1, angiopoetin-1; SHh, Sonic Hedgehog; GDNF, glial cell line-derived neurotrophic factor; FGF, fibroblast growth factor; IGF-1, insulin-like growth factor-1; ApoE, apolipoprotein E.

Astrocytes are essential components of the CNS. These cells are fundamental to the integrity and function of the BBB by regulating its stability and selectively mediating molecular exchange between the bloodstream and the brain parenchyma [80]. Additionally, astrocytes contribute to the stability of the BBB by directly interacting with endothelial cells and pericytes, providing structural support through specialized extensions called astrocytic end-feet, modulating TJ proteins, and actively responding to injury or barrier disruption (Figure 2) [7].

Astrocytes interact with other cells to maintain the integrity of the BBB. Endothelial cells are considered the primary structure of the BBB, and astrocytes support their function by releasing signaling molecules, such as TGF-β, glial cell line-derived neurotrophic factor (GDNF), and angiopoetin-1 (ANG-1), among others, which stabilize TJs between endothelial cells, thus preserving the impermeability of the barrier (Figure 2) [81]. Astrocytes also play a crucial role in the neurovascular unit by communicating with pericytes, which is essential for regulating capillary blood flow, ensuring vascular stability, and maintaining BBB integrity. This intricate communication is mediated by various signaling pathways, with a particularly important pathway involving platelet-derived growth factor (PDGF). PDGF, particularly PDGF-BB, a potent isoform of the PDGF family, is secreted by astrocytes and acts on pericytes by binding to their PDGF receptors, PDGFR-β, triggering a cascade of multiple intracellular signaling events. This astrocyte–pericyte interaction via the PDGF-BB/PDGFR-β axis plays a crucial role in several processes, including the recruitment of pericytes to developing blood vessels, ensuring proper pericyte coverage, maintaining pericyte population (supporting pericyte survival and differentiation), and facilitating the attachment of pericytes to endothelial cells [82]. Together, these interactions create a dynamic and coordinated network that is essential for the proper function of the BBB.

Astrocytes possess specialized end-feet that cover virtually the entire surface of cerebral capillaries [59], establishing a direct interface between the vascular compartment and the neuroglial cells in the NVU. These end-feet have a high density of intramembranous organic anion transporters (OAPs) [83], water channel aquaporin (AQP)-4, and the ATP-sensitive inward rectifier potassium channel Kir4.1, which are involved in ion and volume regulation [64]. Aquaporin-4 is anchored to the membrane through a scaffolding protein complex known as the dystrophin-associated protein complex (DAPC), which links the astrocytic end-feet to laminin and agrin in the basement membrane (agrin accumulates in brain microvessels at the time of BBB tightening and is important for maintaining the integrity of the barrier [47,84]). Additionally, astrocytic end-feet contain other important structures, including microtubules, mitochondria, and intermediate filaments composed of glial fibrillary acidic protein (GFAP) [85]. They also house proteins involved in blood flow regulation, such as phospholipase A2, phospholipase D2, cyclooxygenase 1 (COX1), and prostaglandin E2 synthase [86,87,88].

Astrocytes within the NVU are responsible for secreting several key molecules that support BBB integrity. These include ANG-1, Sonic Hedgehog (SHh), retinoic acid, Wnt growth factor, GDNF, fibroblast growth factor (FGF), insulin-like growth factor-1 (IGF-1), and apolipoprotein E [7]. These molecules play crucial roles in regulating vascular stability, neuroprotection, and the maintenance of BBB function. However, it is important to note that, in neurological disorders, astrocytes are also able to release molecules such as VEGF, matrix metalloproteinases, glutamate, nitric oxide, and endothelins, which, when overexpressed or dysregulated, increase BBB permeability, compromising its ability to block the entrance of toxic substances [7].

Angiopoetin-1 is a glycoprotein secreted by astrocytes and endothelial cells and is responsible for decreasing the permeability of endothelial cells by promoting the expression of junctional proteins, thereby enhancing the integrity of the BBB. This molecule binds to the Tie-2 tyrosine kinase receptor expressed on endothelial cells, activating signaling pathways such as phosphoinositide 3-kinase (PI3)/protein kinase B (Akt), Ras, and mitogen-activated protein (MAP) kinases. These pathways are crucial for endothelial cell repair mechanisms and play a protective role in maintaining BBB integrity [89]. Upon binding of ANG-1 to the receptor protein tyrosine kinase (Tie-2), the adaptor protein Src homology-2-domain protein tyrosine phosphatase-2 is recruited, leading to the downstream stimulation of the extracellular signal-regulated kinase (Erk)1/2 via growth factor receptor-bound protein 2 (Grb2). This signaling pathway suppresses VEGF-induced expression of intercellular adhesion molecule 1 (ICAM-1) and vascular cell adhesion molecules (VCAMs), thereby reducing permeability [90,91]. Overall, ANG-1 inhibits VEGF-induced endothelial permeability by reducing Src-mediated phosphorylation of VE-cadherin. Additionally, ANG-1 is also capable of upregulating the expression of occludin and ZO-1, key TJ proteins, further contributing to the maintenance and integrity of the BBB [92].

Astrocytes are also capable of secreting SHh, while endothelial cells express high levels of the SHh receptor patched-1 (PTCH-1), the signal transducer smoothened (Smo), and transcription factors of the Gli family. These components are all essential for the proper functioning of SHh. Sonic hedgehog binds to the transmembrane PTCH-1 receptor, suppressing the inhibition of Smo. Consequently, this activation allows Gli transcription factors to be transported to the nucleus, where they promote the transcription of SHh target genes [93]. This process induces the expression of junctional proteins, including claudin-3, claudin-5, occludin, JAM-A, VE-cadherin, and ZO-1, thereby promoting BBB integrity. Additionally, SHh has also been shown to reduce the levels of microglial pro-inflammatory cytokines, such as Tumor necrosis factor-alpha (TNF-α), interleukin-6 (IL-6), and interleukin-1 beta (IL-1β) [94].

Wnt growth factors and the Wnt/β-catenin pathway also play a crucial role in regulating BBB integrity. The Wnt/β-catenin pathway is initiated when Wnt growth factors bind to Frizzled (Fzd) and low-density lipoprotein receptor-related protein (LPR) 5 or 6. This binding of Wnt to the receptor promotes the recruitment of disheveled protein and the inhibition of the β-catenin phosphorylation complex [composed of axin, glycogen synthase kinase 3 beta (GSK3β), adenomatous polyposis coli, and casein kinase-1]. The inhibition of this β-catenin phosphorylation complex allows β-catenin translocation to the nucleus, promoting the transcription of target genes involved in vascular integrity, angiogenesis, and TJs. Additionally, increased levels of β-catenin facilitate the expression of claudin-3 and claudin-5 in endothelial cells, reducing BBB permeability [95].

Retinoic acid, a metabolite of vitamin A, is highly expressed in reactive astrocytes (astrocytes that undergo morphological and functional changes in response to CNS injury) and increases the expression of ZO-1 and VE-cadherin, while decreasing the expression of VCAM-1 in brain endothelial cells [96,97]. These effects strengthen TJs between endothelial cells, enhancing the functionality and reducing the permeability of the BBB. Therefore, this mechanism protects the brain against toxic substances and inflammatory mediators, while promoting the overall stability of the neurovascular unit, particularly during pathological conditions such as injury or disease.

GDNF is a neurotrophic factor that has a fundamental role in neuronal survival, growth, and differentiation [98]. GDNF also promotes the expression of occludin, claudin-5, and ZO-1, demonstrating protective effects on the BBB through the upregulation of TJ proteins [99]. Furthermore, FGF, especially FGF-2, is also important in maintaining BBB integrity. FGF binds to its receptor, FGFR, activating downstream signaling pathways, including phospholipase C gamma (PLCγ), ERK, and signal transducer and activator of transcription 3 (STAT3), which regulate several cellular processes. Additionally, FGF also promotes the expression of TJ proteins (claudin-5, occludin, and ZO-1) and the release of GDNF and VEGF, further reinforcing BBB integrity [100,101].

In response to injury or disease, astrocytes also play a critical role in repairing and modulating inflammation at the BBB. As previously mentioned, astrocytes release molecules to support BBB integrity in an attempt to counteract barrier damage and promote re-establishment of TJs [102]. Additionally, during inflammatory injury, these cells also release both pro- and anti-inflammatory cytokines to regulate immune cell activity and mitigate further BBB damage. By balancing these pro- and anti-inflammatory actions, astrocytes help to prevent chronic inflammation, which could otherwise lead to sustained BBB disruption and subsequent neuronal damage [103]. In any case, astrocytes exhibit plasticity in response to injury, which allows the replacement of damaged astrocytes and the maintenance of vascular coverage [104].

Overall, astrocytes are fundamental to the integrity and functionality of the BBB, achieving a wide range of functions, including direct interactions with endothelial cells and pericytes, secretion of key signaling molecules, and structural support via their specialized end-feet. These cells also release anti-inflammatory molecules and growth factors that promote endothelial regeneration and re-establishment of TJs, thereby restoring BBB function. The dynamic interactions between astrocytes and other cells of the NVU highlight their essential role in maintaining the BBB’s integrity and the overall stability of the central nervous system, especially during pathological conditions.

## 4. Molecular Motors and Their General Role in Intracellular Transport

**Figure 3:**
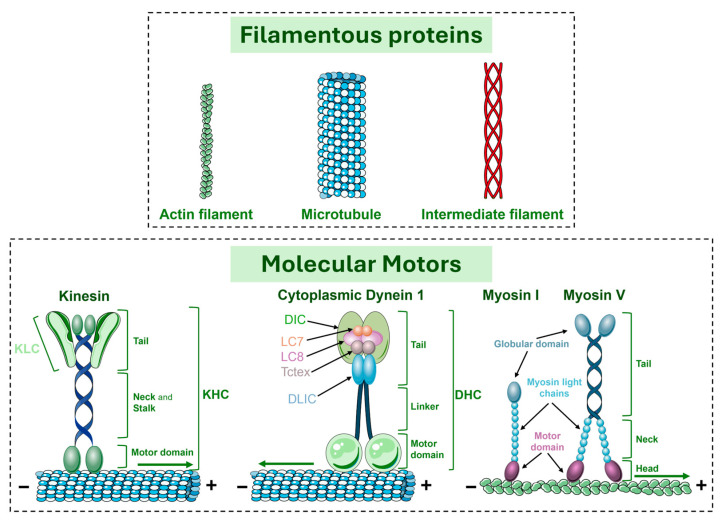
Schematic representation of the primary groups of cytoskeletal filament proteins and their associated molecular motors. (**Top**): The cytoskeleton is composed of actin filaments, microtubules (formed from α- and β-tubulin subunits), and intermediate filaments, which provide structural support and intracellular transport pathways. (**Bottom Left**): Kinesin structure. Kinesins are microtubule-associated motor proteins responsible for transporting cargo toward the plus ends of microtubules (with the exception of kinesin-14, which moves toward the minus ends, and kinesin-13, which does not engage in directional transport but instead regulates microtubule dynamics). They typically function as dimers, each composed of kinesin heavy chains (KHCs) with motor domains at the N-terminal ends. A neck linker connects these motor domains to the stalk region, and the tail region facilitates the binding of kinesin light chains (KLCs) and cargo. (**Bottom Center**): Cytoplasmic dynein 1 structure. Dynein is the primary motor protein that transports cargo toward the minus ends of microtubules. The dynein complex consists of a dimer of dynein heavy chains (DHCs), which generate force for movement. The structure is further stabilized by dimers of dynein intermediate chains (DICs) and dynein light intermediate chains (DLICs) that associate with the DHCs. Additionally, dimers from the three dynein light chain (DLC) families, Roadblock (Robl/LC7), LC8, and Tctex, bind to the N-terminal regions of the DICs. (**Bottom Right**): Myosin I and myosin V structures. Myosins are actin-based motor proteins, with most (except myosin VI) moving toward the plus ends of actin filaments. Myosin I functions as a monomer, while conventional myosins, including myosin V, operate as dimers. Both myosin types typically include a motor domain that generates force, a neck region that binds myosin light chains, and a tail domain that interacts with cargo (myosin V) or the membranes (myosin I) or organelles (as observed with myosin V).

The cytoskeleton is a dynamic network of protein filaments that extends throughout the cytoplasm of cells. This network provides mechanical support for cells to maintain their overall integrity and perform their functions. The eukaryotic cytoskeleton is composed of three main classes of filamentous proteins: microtubules, actin, and intermediate filaments (Figure 3). Each type of cytoskeletal polymer exhibits a distinct arrangement of its protein subunits [105].

Microtubules are composed of α- and β-tubulin dimers that assemble into hollow cylindrical structures [106]. Since α-/β-tubulin heterodimers are intrinsically asymmetric, microtubules are polarized structures with two distinct ends: slowly dynamic minus ends with exposed α-tubulin monomers and highly dynamic plus ends with exposed β-tubulin monomers that grow and shrink through a process designated as dynamic instability [106]. Microtubules play fundamental roles in many cellular functions, such as providing structural support for the cell and assembly of mitotic spindles, and, together with actin filaments, they work as tracks for intracellular transport and organelle distribution [105,107].

Actin filaments are composed of actin subunits arranged in a polarized helical structure. Such an organization can be described as two parallel, right-handed strands offset by half a subunit, or as a short-pitch, left-handed helix, where a single strand connects each subunit to the next one in the opposite strand. Once all asymmetric actin subunits within the polymer are oriented in the same direction, the filament has an inherent polarity, with two distinct ends: a fast-growing barbed end and a slow-growing pointed end. Actin filaments are essential for a variety of functions: intracellular transport, cytokinesis, endocytosis and exocytosis, maintenance of cell shape, and motility [105].

Intermediate filaments are a group of mid-sized filaments (~10 nm in diameter) whose composition is highly variable, depending on the cell type and function. Their main function is to provide mechanical support to the cytoplasm, the cell surface, and the other cytoskeleton proteins. Intermediate filaments do not serve as tracks for molecular motors, yet they can be transported by them [105,108].

Intracellular transport is performed by molecular motor proteins. They include kinesins and dyneins, which interact with microtubules, and myosins, which operate along actin filaments [12]. Molecular motor proteins use the chemical energy from ATP to generate mechanical force. Specifically, when ATP binds to the motor protein, it causes an initial conformational change that detaches the motor protein from the track (actin filaments or microtubules). Then, ATP hydrolysis into adenosine-5′-diphosphate (ADP) and inorganic phosphate (Pi) releases energy, causing a major conformational change in the structure of the motor protein, resulting in its reattachment to the track. This generates force, moving the protein along the microtubules or actin filaments. The binding of a new ATP molecule enables the motor protein to detach again from the track, allowing the cycle to repeat [12].

### 4.1. Kinesin Motors

The first kinesin was described in 1985 by Vale and colleagues while investigating the mechanisms of axonal transport in squid giant axons. They used an in vitro assay to demonstrate that a previously unknown motor protein could move vesicles along microtubules in the presence of ATP [109]. This motor, now referred to as kinesin-1, was for many years the only known molecular motor responsible for transport toward the plus end of microtubules. Remarkably, numerous kinesin-related proteins were identified in the early 1990s, shortly after the sequence of the first gene encoding a kinesin-1 heavy chain (*Drosophila melanogaster* Khc) was made publicly available in genetic databases [110]. Currently, kinesins are a large and diverse superfamily of microtubule-based motors with up to 45 members in humans, divided into 15 families, which are termed kinesin 1 to kinesin 14B, according to phylogenetic analyses [12,111,112]. These motors are evolutionarily conserved and are characterized by their movement toward the plus ends of microtubules (anterograde movement; with the exception of the kinesin-14 family, which moves in the opposite way) [12,13,15,113].

Kinesins possess two distinct functional domains: a conserved motor domain (also known as the “head”), which hydrolyses ATP and generates force and motility in microtubules, and a divergent tail domain that facilitates oligomerization and cargo recognition (Figure 3). With a relatively small and conserved head fused to a diverse array of tail domains, kinesins are able to bind to various types of cargo. Despite the significant sequence similarity found among motor domains, not every kinesin acts as a molecular motor. For example, kinesin-13 is implicated in the regulation of microtubule dynamics [12,110]. These divergent structural features and motility mechanisms of kinesins underlie their diverse cellular functions [110].

Kinesins perform a range of cellular functions, including transporting vesicles and organelles, regulating spindle dynamics during mitosis and meiosis, and maintaining the cytoskeletal structure. In neurons, kinesins play a crucial role in axonal growth and branching, dendrite differentiation, and neuronal migration [14]. Genetic variants of kinesin genes (KIF genes) are linked to neurodegenerative disorders. For example, mutations in KIF1Bb cause the human peripheral neuropathy Charcot–Marie–Tooth disease type 2A (CMT2A) [114].

### 4.2. Dynein Motors

Initially identified as an ATPase in Tetrahymena pyriformis cilia [115], dynein was named after the unit of force the dyne (dyne, force; -in, protein) [116]. This ATPase is an axonemal-specific dynein whose function is to power the motion of motile cilia and flagella, and it is currently known as axonemal dynein. In 1987, a cytoplasmic form of dynein was isolated from the bovine brain by Richard Valle’s lab, when the microtubule-associated protein 1C (MAP1C) was identified as a microtubule-activated ATPase capable of driving intracellular transport towards the minus ends of microtubules [117]. There are two types of cytoplasmic dynein: cytoplasmic dynein-1, which plays several cellular roles, and cytoplasmic dynein-2 [also called intraflagellar transport (IFT) dynein], which only plays a role in transporting cargo along motile and sensory cilia and flagella. Additionally, there is a specific axonemal dynein whose function is to power the beating of motile cilia [16]. The human genome comprises 16 different dynein heavy chain (*DHC*)-encoding genes, 14 of which encode axonemal dyneins and only 2 of which encode cytoplasmic dyneins [16,118]. Cytoplasmic dyneins are present in nearly all eukaryotes, except for red algae, flowering plants, and anaerobic parasites of the genus Entamoeba [119].

Cytoplasmic dynein 1 (hereafter called dynein) is composed of multiple protein complexes assembled around a dimer of force-generating subunits named DHCs (Figure 3). Each DHC contains one motor domain attached to a divergent N-terminal tail domain. The motor domain comprises an AAA+ ring which powers the motor by converting chemical energy from ATP hydrolysis into mechanical force, driving dynein’s movement along microtubules. Additionally, it has a microtubule-binding domain which is responsible for attaching dynein to the microtubule track. The tail domain is important for oligomerization, acting as a scaffold for the binding of other dynein subunits. These subunits facilitate the connection of dynein to various cellular cargos, either through direct binding or by recruiting adaptor proteins that mediate specific cargo interactions [15,16].

Dynein is functionally regulated by three important cofactors: dynactin, lissencephaly 1 (LIS1), and Nuclear Distribution Protein E (NudE) and its homolog NudE-Like (NudE/L). These cofactors are, for instance, relevant for the initiation and activation of dynein’s motility and its localization to microtubules plus ends [15,120]. Besides these important cofactors, dynein is regulated by a group of proteins termed activating adaptors, which are important in establishing dynein and dynactin interactions and binding to cargo [15].

Dynein is a fundamental protein involved in numerous cellular functions, including organelle positioning, vesicular transport, protein and mRNA transport, axonal growth, chromosome dynamics, nuclear envelope breakdown, mitotic spindle assembly and positioning, and cell migration, among others [12,15,16]. Dysfunctions in dynein-mediated transport are increasingly associated with neurodevelopmental disorders, such as lissencephaly, as well as neurodegenerative diseases, including spinal muscular atrophy with lower extremity predominance (SMA-LED) [15]. Moreover, mutations in dynactin’s subunit p150Glued, coded by the gene *DCTN1*, are associated with a rare neurodegenerative disease, Perry syndrome. Additionally, rare genetic variants in *DCTN1* have been associated with other neurodegenerative disorders, such as amyotrophic lateral sclerosis (ALS), frontotemporal dementia (FTD), and syndromes resembling progressive supranuclear palsy (PSP). These findings underscore the role of the dynein–dynactin motor complex in diverse neurodegenerative conditions [121].

### 4.3. Myosin Motors

In 1864, Wilhelm Kühne first described myosin as a viscous protein extracted from skeletal muscle, proposing that it was responsible for maintaining the muscle’s tension state. This discovery was pivotal in laying the foundation for the study of muscle biochemistry and contractility [122]. Unlike kinesin and dynein, myosins are a superfamily of actin-based motor proteins that bind and translocate actin filaments (F-actin) through mechanical forces. Most myosins move toward the actin barbed end [12].

The myosin superfamily is a large and diverse group of proteins, organized into 18 classes, involved in a variety of cellular pathways [17]. Structurally, most myosins are dimers and are characterized by three main regions: the head, the neck, and the tail (Figure 3). The head region comprises the motor domain, and each myosin can have one or two heads at its N-terminus. The motor domain directly binds actin filaments and powers myosin’s movement along filaments through its ATPase activity. The neck is an elongated region of variable length that is connected to each myosin head. Its main role is to regulate myosin movement. The tail at the C-terminus is the most divergent region and plays a critical role in complex dimerization and in specific recruitment. The tail determines whether a myosin is single-headed (monomer) or two-headed (dimer). Depending on the class, myosin motors may include a versatile globular region known as the cargo binding domain (CBD), along with structural motifs like coiled-coil regions that facilitate motor assembly [123,124,125]. Myosin motor function is directly regulated by phosphorylation via kinases, such as Rho-associated kinase (ROCK) and myosin light chain kinase (MLCK), or indirectly via inhibition of myosin light chain phosphatase (MLCP) by ROCK. Myosin and ROCK are downstream effectors of ras homolog family member A (RhoA) guanosine-5′-triphosphate (GTP)ases, key players in cytoskeletal dynamics [126,127].

Myosins are generally divided into two major groups: conventional myosin-II and unconventional myosins. Conventional myosin-II is best known for its role in muscle contraction and cytokinesis. In contrast, unconventional myosins, including myosin-I, -V, and -VI, encompass a wider range of intracellular functions, such as cargo transport, cell motility, endocytosis, and exocytosis. All myosin motors move toward the plus ends of actin filaments, except for myosin-VI, which uniquely moves toward the minus ends [128].

Myosins are essential for all contractile-dependent cellular and tissue processes, such as muscle contraction, cytokinesis, and cell motility. In addition, myosins also perform short-range transport, often close to the plasma membrane [17]. Mutations in genes coding for myosin subunits are related to a variety of human diseases and syndromes. For instance, genetic alterations in myosin-II can lead to hypertrophic cardiomyopathy, a malformation and dysfunction of the heart, and mutations in Myosin-Va are associated with Griscelli syndrome, which is characterized by defects in pigmentation and neuronal malfunction [17].

## 5. Role of Microtubules and Molecular Motors in the Trafficking of Junctional Proteins

The importance of molecular motors in maintaining cell junction homeostasis was first revealed in studies using the microtubule-disrupting drug colchicine. Specifically, Madin–Darby canine kidney (MDCK) cells treated with colchicine exhibited changes in TEER values, a measure of TJ barrier function, as well as structural alterations in TJs, as observed by electron microscopy [129].

In human intestinal cells, the formation of the TJ barrier depends on the integrity of microtubules and dynein [130], suggesting that minus-end-directed transport is involved in delivering TJ proteins to the membrane. Further evidence of the role of microtubules in TJ formation comes from findings that treatment with nocodazole (which interacts with β-tubulin and interferes with the dynamics of microtubule assembly and disassembly [131]) or depletion of Dual leucine zipper-bearing kinase (DLK), which is necessary for microtubule reorganization, significantly reduces ZO-1 and claudin levels at the cell periphery in keratinocytes [132,133]. On the other hand, the microtubule-stabilizing drug paclitaxel promotes the accumulation of junctional proteins at the cortex and helps barrier formation in reconstructed human epidermis [133]. Consistent with this, recent studies have confirmed that occludin moves along microtubule tracks [130].

Despite their role in organizing and maintaining junctional integrity, microtubules also participate in the disassembly of TJs [134]. In the absence of the microtubule-depolymerizing drug nocodazole, Ca^2+^ depletion causes junction breakdown, the formation of intracellular contractile actin rings, and the internalization of TJ proteins like occludin and ZO-1, along with AJ proteins such as E-cadherin and β-catenin, which become associated with these actin structures [134]. When microtubules are depolymerized with nocodazole, this movement of junctional proteins into the cytosolic actin rings is blocked [134]. On the other hand, stabilizing microtubules with drugs reduces the disassembly of apical junctions and prevents actin ring contraction. Microtubule-associated motor proteins are also involved in actin ring formation, as Kinesin-1 has been shown to localize at junctions and associate with E-cadherin and catenins in epithelial cells [134].

Actomyosin contractility is also essential for junction disassembly after Ca^2+^ depletion. The RhoA-ROCK signaling pathway, driven by the microtubule-associated guanine exchange factor H1 (GEF-H1), is involved in actin ring formation and contraction after Ca^2+^ loss, indicating that microtubules control junction disassembly by regulating actomyosin contractility [135]. Additional evidence supporting the involvement of microtubules in the stability of TJs and AJs comes from findings that overexpression of β-tubulin chaperone cofactor D, a GTPase activating protein for β-tubulin, disrupts junction assembly and prevents their formation in MDCK cells [136].

Other studies with thyroid epithelial cell monolayers have also shown that the disassembly of microtubules with colchicine decreased the accumulation of E-cadherin at the junctions [137].

On the other hand, studies have shown that AJ proteins are transported to the cell surface via the dynamic plus ends of microtubules and their associated motor proteins [138]. In fibroblasts, kinesin is essential for establishing N-cadherin-dependent cell–cell interactions [139], and the association of p120-catenin with kinesin facilitates the transport of the catenin–cadherin complex to the junctions [140]. In support of this, the overexpression of Kinesin family member 17 (KIF17) enhances the junctional accumulation of E-cadherin [141]. In epithelial Pkt2 cells, cytoplasmic dynein tethers microtubules at cell–cell contact sites during the formation of junctions, serving as a pathway for kinesin-mediated delivery of junctional components [142].

Non-muscle myosin was also found at the apical junctions of epithelial cells, forming sarcomere-like structures organized in a belt configuration, which is crucial for preserving normal junctional tension [143]. However, such a structure has yet to be identified in brain endothelial cells, despite indications that non-muscle myosin is associated with the TJ complex in these cells [144].

## 6. Molecular Motors in Mitochondrial Transport and Energy Supply in Astrocyte Processes

One of the primary energy needs of the BBB comes from its reliance on active transport mechanisms. Essential nutrients, such as glucose and certain amino acids, are actively transported from the bloodstream into the brain. This process is mediated by specific transporters located on the membranes of endothelial cells which utilize ATP to drive the uptake of these crucial molecules against their concentration gradients [145].

Due in part to their small size, it was initially considered that astrocyte processes could not contain mitochondria. Nevertheless, studies have shown the presence of mitochondria within astrocytic projections [146,147,148] and that astrocyte end-feet are highly specialized regions that have a high energetic demand [149]. In line with this, a large portion of the astrocyte proteome is dedicated to perivascular end-feet, with particular enrichment in proteins involved in oxidative phosphorylation [146].

Previous studies have shown that the movement of mitochondria within astrocytes is reduced following vinblastine (an inhibitor of tubulin polymerization) and cytochalasin D (an inhibitor of actin polymerization) treatment, indicating that mitochondrial trafficking in astrocytes is dependent on microtubule and actin networks [149,150]. This also suggests that the transportation and allocation of mitochondria to active areas within astrocytes are likely regulated by their interactions with molecular motors. In neurons, the targeted movement of mitochondria primarily occurs by kinesins and dynein along the microtubule networks [151,152,153,154]. Particularly, in neuronal axons, the majority of anterograde transport occurs through kinesin motors, whereas retrograde transport relies on dynein. In dendrites, due to the presence of mixed microtubule polarity, both kinesin and dynein motors can transport mitochondria in both directions [152]. In astrocytes, the orientation of microtubules remains unclear. However, studies have shown that mitochondrial transport in astrocyte processes is slower than in neurons [147] but occurs at comparable speeds and with similar ratios during both anterograde and retrograde transport [147,149], potentially suggesting a mixed orientation similar to that found in neuronal dendrites. Moreover, in comparison to neurons, a smaller fraction of mitochondria are motile in astrocyte projections [149]. Despite this, while in neurons several members of the kinesin family, including kinesin-related protein 5 (KIF5; kinesin-1), have been implicated in mitochondrial movement [153,155,156], it remains unclear which kinesin motors are responsible for mediating mitochondrial transport in astrocytes. It is, however, known that an elevation in astrocytic intracellular Ca^2+^ levels triggers Miro1 EF-hand-dependent mitochondrial location at synapses [147]. In neurons, Ca^2+^ binding to the EF-hand also allows mitochondrial localization at synapses [157,158]. Considering that Miro proteins link mitochondria to KIF5 [154,157,158,159], this indicates that these motor proteins may play a role in moving mitochondria along the microtubules within astrocyte processes.

On the other hand, at resting (low) Ca^2+^ levels, disrupting actin filaments did not affect baseline mitochondrial movement [150]. However, after an increase in intracellular Ca^2+^ levels, actin filament disruption led to an increase in the proportion of motile mitochondria in astrocyte processes [150]. This suggests that Ca^2+^ also regulates mitochondrial movement in astrocytes by modulating their connection with actin filaments.

Beyond transport, there is also the exchange of mitochondria between astrocytes and neurons. For instance, astrocytes can deliver healthy mitochondria to injured neurons, helping in neuroprotection and repair [160,161]. After stroke, astrocytes were shown to secrete extracellular mitochondrial particles through cluster of differentiation 38 (CD38; also known as cyclic ADP ribose hydrolase)-dependent pathways, which were then taken up by neurons [160]. Similarly, astrocytic mitochondria were detected in cisplatin-damaged neurons, an effect that was prevented following Miro-1 knockdown in astrocytes. This indicates that the mitochondrial adapter Miro-1 is essential for facilitating mitochondrial transfer from astrocytes to neurons affected by cisplatin, likely involving transport via molecular motors [161]. Nevertheless, the mechanistic details behind this mitochondrial transfer from astrocytes to damaged neurons remain unclear.

On the other hand, astrocytes can also absorb damaged mitochondria from neurons [162]. Particularly, axonal protrusions containing damaged mitochondria at points of contact with astrocytes, along with axonal evulsions enveloped by astrocytes and containing mitochondria, allowed mitochondria transfer to astrocytes to undergo degradation. This process, termed transmitophagy, was described by Davis and colleagues at the optic nerve head [162], challenging the general assumption that neurons degrade their own mitochondria. However, the specific mechanisms underlying the transfer of damaged neuronal mitochondria to astrocytes for degradation remain uncharacterized. Therefore, it would be interesting to further explore whether a similar exchange of mitochondria occurs between astrocytes and neurons (or other cells) surrounding (or forming) the BBB.

## 7. Molecular Motors in the Regulation of Water and Ion Homeostasis via Aquaporin-4

Aquaporins are a group of specialized water channel proteins characterized by their ability to facilitate rapid and selective water transport across cell membranes [163]. Mammalian cells express 13 different AQP proteins. Among them, AQPs 0, 1, 2, 4, 5, 6, and 8 are solely water-permeable, while AQPs 3, 7, 9, and 10 can also transport small solutes like urea and glycerol [164]. Unlike other aquaporins, AQP-4 is expressed abundantly in the brain, especially in astrocytes [165,166]. There are two major isoforms of AQP-4, AQP-4a (M1) and AQP-4c (M23), which result from alternative splicing [167]. These isoforms form heterotetramers and larger supramolecular structures called orthogonal arrays of particles, whose formation, driven primarily by the M23 isoform, is critical for the functional clustering of AQP-4 at astrocytic end-feet [168,169].

The highly polarized distribution of AQP-4 at astrocyte end-feet, which are in direct contact with blood vessels and the pia mater, highlights its critical role in regulating water homeostasis across the BBB [170]. Thus, the transport of water mediated by AQP-4 contributes to the regulation of intracranial pressure, osmoregulation, and the clearance of metabolic waste via the glymphatic system [171].

Studies have shown that microtubules are crucial for targeting AQP-4 at the basolateral plasma membrane in epithelial cells [172]. In MDCK cells, disruption of the microtubule network with nocodazole decreased the fraction of motile vesicles containing green fluorescent protein (GFP)-AQP-4, supporting a role for microtubules in protein transport [172]. However, the disruption of F-actin fibers with cytochalasin D had no effects on the dynamics of GFP-AQP-4 vesicles, suggesting that they do not use the actin cytoskeleton for intracellular distribution [172].

Since movement along microtubules depends on molecular motors, the efficient localization of AQP-4 to astrocytic end-feet likely involves intracellular trafficking pathways driven by these complexes. Supporting this hypothesis, Alzheimer’s disease, which is characterized by defects in the transport of intracellular components by motor proteins [173], also exhibits reduced localization of AQP-4 at astrocytic end-feet [174]. Overall, this indicates that the targeting of AQP-4 to astrocyte end-feet, where it regulates water and ion homeostasis, is likely mediated by molecular motors.

## 8. Myosin Motors in Astrocyte Morphology, Actin Remodeling, and Migration

To support neuronal function, astrocytes must dynamically regulate their morphology, enabling migration and the formation of stable adhesions to brain vascular tissues. While the molecular pathways governing this modulation are still being explored, the actomyosin cytoskeleton is recognized as a key effector driving the mechanical forces behind these processes.

Different myosin isoforms and their regulators are present in cultured astrocytes of different species [14,175,176,177,178]. However, the precise role and activation state of myosin in astrocyte process growth and within end-feet remain unclear. The formation of astrocyte processes that give rise to an astrocyte characteristic stellar morphology and that terminate in astrocyte end-feet requires the modulation of actin and myosin, as well as adhesion to a substrate [179,180]. Notably, myosin inactivation (via inhibition of upstream regulators) is necessary to promote astrocyte process outgrowth. This process is accompanied by the depolymerization of cortical F-actin and their reorganization at the tips of the processes, indicating that myosin must release the tension in the actin network to enable its remodeling and reorganization [179,180,181]. While the localization of myosin in these structures has yet to be fully characterized, proteomic analyses of astrocyte end-feet have confirmed the presence of several myosin isoforms [85,146]. It is possible that myosin must remain inactive during the growth phase of astrocyte processes but become activated upon contact with endothelial cells, where this activation could generate critical traction forces to stabilize the end-feet and ensure strong adhesion to endothelial cells.

The mechanical forces exerted by myosin on actin also seem to play a paradoxical role depending on the cellular context: they may stabilize actin in focal adhesion sites during cell stationary states or drive cellular contraction during migration.

Under normal physiological conditions, astrocytes generally remain stationary to maintain stable connections with the vascular tissue [182]. Some evidence suggests that myosin pathways must be active to maintain astrocyte immobility. Yet myosin also appears to play a crucial role in enabling cell motility. Studies in cultured astrocytes have shown that inhibiting the myosin upstream regulators ROCK and Rho GTPase enhances astrocyte migratory activity [181,183]. In human astrocytoma cells, disruption of myosin activity using low concentrations of a chemical inhibitor also enhances their migratory behavior [184]. In contrast, studies using similarly low concentrations of the same myosin chemical inhibitor in cultured astrocytes reduced cell migration [182]. At higher inhibitor concentrations, where myosin function is completely abolished, both astrocyte and astrocytoma cell migration are inhibited [182,184]. The contrasting results may stem from inherent differences in the cell lines used, variations in the duration of chemical treatments, or differing experimental conditions. Nevertheless, these data suggest that myosin requires a finely tuned regulation to balance its functions in astrocytes. In support of this hypothesis, evidence suggests that in the case of a lack of myosin activation, traction forces and extracellular matrix (ECM) tension are reduced, destabilizing adhesions and causing morphological changes, such as cell rounding, which may serve as a critical switch to a migratory phenotype. However, the process of migration itself requires myosin activation to generate the contractile forces and transient adhesions necessary for movement [185,186]. Ultimately, these findings support the hypothesis that astrocytes rely on an actomyosin-driven crawling mechanism to migrate, as observed in in vitro two-dimensional (2D) models [187,188].

In pathological conditions, such as neuronal injury, degeneration, and neuroinflammation, astrocytes detect mechanical and chemical signals and change their phenotype into a reactive state, migrating toward the affected site. These astrocytes can gradually turn into scar-forming astrocytes. This transformative process is called astrogliosis [189,190]. Myosin’s role in astrogliosis is also under debate. Studies report that the Rho GTPase-ROCK pathway is upregulated in reactive astrocytes following brain injury, and this is strongly associated with glial scar formation [191]. A glial scar consists of a dense mass of reactive astrocytes with interwoven processes and secreted ECM components, such as collagen, with contributions from other molecules and glial cells. This scar forms a protective barrier that isolates damaged tissue but also limits axonal regeneration [189,190]. Since ROCK promotes myosin activity and F-actin stabilization, it is likely that myosin also becomes overly activated during scar formation, driving the generation of compact actin structures, such as stress fibers, which contribute to the establishment of a glial scar. In line with this, the inhibition of the Rho GTPase-ROCK-myosin pathway was shown to reduce the astrocyte reactivity phenotype [191]. Furthermore, possibly due to reduced astrogliosis and scar formation, the inhibition of this pathway has been shown to result in axon growth, potentially improving neuronal regeneration after injury [192].

Conversely, other studies suggest that inactivation of the Rho GTPase-ROCK-myosin pathway promotes astrogliosis and fails to promote axon regeneration following brain injury in vitro and in vivo. Myosin inhibition results in a reactive astrocyte phenotype similar to that observed with Rho GTPase-ROCK inhibition, including alterations in cortical F-actin organization, reduced myosin-mediated actin compaction, and loss of focal adhesions. These activate mechanotransduction signals that drive the increased expression of GFAP, a key marker of reactive astrocytes, and chondroitin sulfate proteoglycans (CSPGs), astrocyte-secreted ECM components of glial scars [79,177,183,193,194]. Altogether, these seemingly contradictory data highlight myosin as a critical factor in astrogliosis, suggesting that myosin activity is likely limited during migration in the initial stages of astrocyte reactivity but becomes active and exerts tension on astrocytes’ actin cytoskeletons during glial scar formation. Nevertheless, it is important to take into account that ROCK has several other cytoskeletal functions, such as increasing cell adhesion, that may contribute to the overall phenotype of astrocytes and glial scar formation [191].

Beyond chemical signaling, physical changes in the microenvironment following brain injury are also major drivers of astrocyte phenotype transformation, with myosin playing a central role. Several in vitro and in vivo studies have demonstrated that astrocytes sense ECM changes through adhesion molecules and respond via feedback mechanisms mediated by myosin activity on the actin cytoskeleton, ultimately modulating their phenotype [79,185,195,196,197].

Overall, these studies indicate that astrocyte actin-dependent processes, such as process formation, immotility, migration, and reactivity are all dependent on myosin’s balanced regulation. Further studies are necessary to clarify the precise mechanisms governing myosin’s role and activation state during these processes.

## 9. Molecular Motor-Driven Cytokine Release and Neuroinflammatory Response

During neuroinflammation, astrocytes both receive and release pro-inflammatory cytokines such as TNF-α and IL-1β, which modulate the response of other immune cells and significantly influence BBB permeability, enabling perivascular leukocyte infiltration [198,199]. While the trafficking and release mechanisms of other astrocyte-secreted proteins, such as neurotransmitters and growth factors, have been described, the release of inflammatory factors, like cytokines, needs further investigation [200].

In other immune cells, such as macrophages and T-cells, cytokine-loaded vesicles are transported and secreted via exocytosis [201,202]. This process typically follows the classical exocytosis pathway, where vesicles are transported from the endoplasmic reticulum (ER)/Golgi complex to the cell membrane via anterograde transport. This directed movement relies on the cytoskeleton as an integrated track system, with microtubules and F-actin playing essential roles [202,203]. Kinesins serve as the primary motor proteins responsible for anterograde transport along microtubules [13], while myosins, which operate on the actin cytoskeleton, also contribute to vesicle trafficking and secretion pathways [17]. In astrocytes, the directional movement of vesicles containing glutamate or other neuroactive substances toward the end-feet for exocytosis has been associated with kinesin motor activity [202,204,205].

Pathological stimuli induce significant cytoskeletal alterations in activated astrocytes and microglia, including increased microtubule dynamics [206], which are associated with facilitated cytokine trafficking and release [207]. Beyond microtubules and actin, IFs are also essential contributors to vesicle mobility in astrocytes [19,208]. Unlike microtubules and actin, IF trafficking does not rely on motor proteins or directionality. Still, IFs are very dynamic in astrocytes and integrate functions with actin and microtubules [19,209]. GFAP, the major IF protein in astrocytes, is a widely used marker for reactive astrocytes and is upregulated in response to pathological stimuli [209,210]. It is possible that GFAP overexpression in reactive astrocytes may also facilitate vesicle trafficking and the release of pro-inflammatory cytokines.

Non-classic pathways that release cytokines have also been described in immune cells and astrocytes, involving membrane channels/transporters rather than the canonical ER/Golgi secretory pathway [211,212,213]. It would be interesting to investigate whether motor proteins play a role in the trafficking and release of cytokines via this alternative secretory pathway in astrocytes.

In the event of BBB disruption, either by exacerbated neuroinflammation or injury, astrocytes release neurotrophic factors aimed at repair, such as brain-derived neurotrophic factor (BDNF), which are primarily secreted in vesicles by exocytosis [200,214]. It is plausible that neurotrophic factor-loaded vesicles are transported to the astrocyte end-feet by kinesin motors [202,204,205]. Meanwhile, dynein motors play an essential role in retrograde transport, retrieving signalling molecules and debris from the perivascular region back to the astrocyte for recycling [202,214]. Therefore, in addition to mediating secretion of pro-inflammatory signals during neuroinflammation, molecular motors also facilitate astrocyte neuroprotective responses aimed at maintaining or re-establishing BBB homeostasis. Impairment of these transport mechanisms can lead to prolonged inflammation, BBB instability, and progression of neurodegenerative diseases [215].

Overall, cytokine and neurotrophic trafficking and release mechanisms in astrocytes are likely similar to neuronal exocytosis and resemble cytokine secretion processes observed in other immune cells. These mechanisms involve the coordinated activity of cytoskeletal motor proteins, such as kinesin, dynein, and myosin, along with additional sorting and membrane-fusion proteins [202]. Despite these parallels, further investigation is necessary to disclose the precise mechanisms and motor proteins governing cytokine-loaded vesicle trafficking in astrocytes to fully elucidate their unique contributions to neuroinflammation and to BBB disruption and repair.

## 10. Conclusions

CNS homeostasis critically depends on the proper functioning of the BBB, a highly specialized structure that safeguards the brain from harmful substances while ensuring the selective transport of essential molecules. Astrocytes play a pivotal role in preserving BBB integrity by secreting key regulatory molecules and neurotrophic factors that influence capillary blood flow, BBB permeability, endothelial cell growth and regeneration, and the inflammatory response. Additionally, astrocytes contribute to BBB ion and water homeostasis via AQP channels and support metabolism by supplying energy, recycling waste, transferring healthy mitochondria, and clearing damaged organelles.

A growing body of evidence underscores the importance of astrocyte cytoskeletal molecular motors in BBB regulation. Kinesin-driven trafficking of junctional proteins along microtubule tracks and actomyosin-driven tension at TJ and AJ sites are both fundamental to BBB integrity. The actomyosin cytoskeleton also supports astrocyte process outgrowth, essential for their stellate morphology and end-feet formation, although the roles, isoforms, and activation states of myosin in these processes remain unclear. Additionally, there is also evidence that mitochondria delivery, AQP-4 distribution, and vesicular trafficking to astrocyte end-feet occur in a microtubule-dependent manner, with contributions from the actin cytoskeleton. However, the specific motors involved and their precise roles in these mechanisms remain to be fully elucidated.

Astrocytes respond to injury, neuroinflammation, and BBB disruption by transitioning into a reactive state, characterized by cytoskeleton-driven morphological changes, migration, and cytokine secretion. Molecular motors also appear to play a crucial role in these processes: kinesin and dynein are associated with the trafficking of cytokine-loaded vesicles along microtubules, while actin-dependent processes, including process formation, adhesion, and migration, rely on finely tuned myosin regulation. However, the specific kinesin and dynein motors involved, as well as myosin’s role and activation state in balancing immotility and migration, remain controversial and warrant further investigation.

Given the importance of molecular motors for cellular homeostasis, their dysfunction can severely compromise BBB integrity, increasing CNS vulnerability. Dysregulation of these motors has been implicated in BBB breakdown, contributing to neurodegenerative diseases through impaired vesicular trafficking, mitochondrial dysfunction, and the loss of endothelial support. Despite their fundamental role, the mechanisms governing several functions of molecular motors in astrocyte-mediated BBB maintenance remain underexplored.

## 11. Future Perspectives

The roles of molecular motors in cellular processes within astrocytes at the BBB, particularly in regulating TJs, mitochondrial transport, and water and ion homeostasis, are exciting and rapidly expanding areas of research. As discussed, microtubules in astrocytes play a crucial role in the trafficking of junctional proteins, mitochondrial distribution, and the functioning of specialized channels like AQP-4. Despite significant advancements in understanding these processes, many critical questions remain unanswered, representing attractive hypotheses for future investigation. One of the most relevant questions is related to the precise molecular mechanisms by which motor proteins coordinate the transport of junctional proteins and organelles within the highly polarized and complex environment of astrocytes that form the BBB. For example, the involvement of RhoA-ROCK signaling in actomyosin contractility during junction disassembly and the regulation of mitochondrial movement in response to Ca^2+^ influx suggest a deep interplay between motor protein activity and cellular signaling networks. Future studies could explore the interplay between motor proteins and signaling molecules, focusing on how alterations in these pathways contribute to disease conditions such as ischemia and neurodegeneration. Additionally, understanding how cells coordinate the directionality of motor-driven transport, particularly in contexts with mixed microtubule polarity, such as in astrocytes, remains a significant research question.

Advanced methodologies, such as super-resolution microscopy and live-cell tracking, are likely to provide more detailed insights into the dynamics of motor protein activity and protein trafficking in real time. These technologies could be particularly useful for investigating mitochondrial transport within astrocyte processes, where slower motility and smaller fractions of motile mitochondria have been observed [149]. Since impaired trafficking and mitochondrial dysfunction are implicated in a wide range of neurological disorders [216,217], understanding the mechanistic basis of their transport within glial cells could reveal novel therapeutic alternatives.

The role of cellular communication between neurons and astrocytes, particularly in the exchange of mitochondria, also requires further exploration. The process of transmitophagy, where damaged mitochondria are removed from neurons and degraded by astrocytes [162], highlights a novel mechanism of cellular cooperation that may be pivotal for BBB preservation and repair. Future studies could elucidate the regulatory mechanisms involved in this pathway, which could have significant implications for understanding the pathophysiology of certain neurodegenerative conditions like Parkinson’s and Alzheimer’s diseases.

Mathematical and theoretical models may also play a crucial role in advancing our understanding of BBB dynamics by using experimental data to predict its behavior, allowing researchers to explore cellular interactions, transport mechanisms, and barrier integrity under physiological and pathological conditions. These approaches could provide new insights into the complex regulatory networks controlling BBB integrity and function and help identify novel therapeutic strategies.

Lastly, since defects in motor protein function underlie numerous brain diseases [173], targeting these complexes with small molecules or gene therapy could provide innovative treatment strategies. Understanding the differentiated roles of motor proteins in astrocyte-dependent maintenance of BBB structure and function will be crucial for designing innovative therapeutic approaches, particularly in conditions where microtubule dynamics are disrupted.

## Abbreviations

The following abbreviations are used in this manuscript:

2D	Two-dimensional
ADP	Adenosine-5′-diphosphate
AJ	Adherens junction
Akt	Protein kinase B
ALS	Amyotrophic lateral sclerosis
ANG-1	Angiopoetin-1
AQP-4	Aquaporin-4
ATP	Adenosine-5′-triphosphate
BBB	Blood–brain barrier
BDNF	Brain-derived neurotrophic factor
CMT2A	Charcot–Marie–Tooth disease type 2A
CNS	Central nervous system
COX1	Cyclooxygenase 1
CSPGs	Chondroitin sulfate proteoglycans
Cx	Connexin
DAPC	Dystrophin-associated protein complex
DHC	Dynein heavy chain
DLC	Dual leucine zipper-bearing kinase
ECM	Extracellular matrix
ER	Endoplasmic reticulum
Erk	Extracellular signal-regulated kinase
FGF	Fibroblast growth factor
FTD	Frontotemporal dementia
Fzd	Frizzled
Gbr2	Growth factor receptor-bound protein 2
GDNF	Glial cell line-derived neurotrophic factor
GEF-H1	Microtubule-associated guanine exchange factor H1
GFAP	Glial fibrillary acidic protein
GFP	Green fluorescent protein
GSK3β	Glycogen synthase kinase 3 beta
GTP	Guanosine-5′-triphosphate
ICAM-1	Intercellular adhesion molecule 1
IFT	Intraflagellar transport
IGF-1	Insulin-like growth factor-1
IL-1β	Interleukin-1 beta
IL-6	Interleukin-6
JAM	Junctional adhesion molecule
KHC	Kinesin heavy chain
KIF5	Kinesin-related protein 5
KIF17	Kinesin family member 17
LIS1	Lissencephaly 1
LPR	Low-density lipoprotein receptor-related protein
M1	Aquaporin-4a
M23	Aquaporin-4c
MAP	Mitogen-activated protein
MAP1C	Microtubule-associated protein 1C
MDCK	Madin–Darby canine kidney
MLCK	Myosin light chain kinase
MLCP	Myosin light chain phosphatase
NudE	Nuclear distribution protein E
NVU	Neurovascular unit
OAPs	Organic anion transporters
PDGF	Platelet-derived growth factor
PI3	Phosphoinositide 3-kinase
PLCγ	Phospholipase C gamma
PSP	Progressive supranuclear palsy
PTCH-1	Sonic hedgehog receptor patched-1
RhoA	Ras homolog family member A
ROCK	Rho-associated kinase
SHh	Sonic hedgehog
SMA-LED	Spinal muscular atrophy with lower extremity predominance
Smo	Signal transducer smoothened
STAT3	Signal transducer and activator of transcription 3
TEER	Trans-epithelial electrical resistance
TGF-β	Transforming growth factor-beta
Tie-2	Receptor protein tyrosine kinase
TJ	Tight junction
TNF-α	Tumor necrosis factor-alpha
VCAM	Vascular cell adhesion molecule
VE	Vascular endothelial
VEGF	Vascular endothelial growth factor
ZO	Zonula Occludens
